# Surgical Considerations of Intractable Mesial Temporal Lobe Epilepsy

**DOI:** 10.3390/brainsci8020035

**Published:** 2018-02-20

**Authors:** Warren W. Boling

**Affiliations:** 11234 Anderson Street, Room 2562-B, Loma Linda, CA 92354, USA; wboling@llu.edu; Tel.: +1-909-558-4479

**Keywords:** temporal lobe epilepsy, selective amygdalohippocampectomy, epilepsy surgery, mesial temporal lobe epilepsy

## Abstract

Surgery of temporal lobe epilepsy is the best opportunity for seizure freedom in medically intractable patients. The surgical approach has evolved to recognize the paramount importance of the mesial temporal structures in the majority of patients with temporal lobe epilepsy who have a seizure origin in the mesial temporal structures. For those individuals with medically intractable mesial temporal lobe epilepsy, a selective amygdalohippocampectomy surgery can be done that provides an excellent opportunity for seizure freedom and limits the resection to temporal lobe structures primarily involved in seizure genesis.

## 1. Introduction

Temporal lobe epilepsy (TLE) affects a substantial number of individuals with medically intractable epilepsy. TLE is the most common operated epilepsy, and the majority of patients with localization related epilepsy seen in tertiary epilepsy centers have TLE [1,2]. Although TLE is not the most common epilepsy. TLE in the general population of Minnesota has been estimated at about 10.4 per 100,000 this is compared with 54.3 per 1000 incidence of epilepsy in the population as a whole [3].

The frequent occurrence of intractable epilepsy in the temporal lobe bears witness to the highly epileptogenic nature of the limbic structures that comprise the mesial portion of the temporal lobe. The mesial part of the temporal lobe is richly connected with surrounding extra-temporal cortical regions especially the orbitomesial frontal lobe via the uncinate fasciculus and the fornix carries fibers from the hippocampus that project to the anteromesial frontal lobe and anterior nucleus of the thalamus [4,5]. The mesial temporal structures are highly connected as well with the anterolateral neocortical temporal lobe. Therefore, due to the strong connections of the mesial temporal structures with the anterior and lateral temporal lobe in addition to other limbic regions, TLE most commonly manifests the semiology of staring and automatisms regardless of the seizure onset zone in lateral or mesial structures of the temporal lobe. 

Despite the frequent occurrence and intractable nature of temporal lobe epilepsy, it responds very well to surgery with high rates of resulting seizure freedom and the risks of surgery are quite low [6,7,8]. The decision to consider a patient with medically intractable TLE for surgery has become much clearer nowadays since the International League Against Epilepsy (ILAE) defined medical intractability as the failure of adequate trials of two tolerated, appropriately chosen and used antiepileptic drugs [9]. A clear definition of intractable epilepsy combined with the excellent results of surgery for symptomatic epilepsy especially arising in the temporal lobe, has resulted in numerous consensus reports affirming that individuals with drug resistant epilepsy should be evaluated in a comprehensive epilepsy program to identify opportunities for surgical cure [10]. 

Temporal lobe epilepsy can be categorized into one of two types based on the anatomical region of seizure onset. Seizures that originate from the temporal cortex lateral to the collateral sulcus are defined as lateral or neocortical epilepsy, and seizures that have a focus of onset medial to the collateral sulcus are named mesial temporal lobe epilepsy (MTLE). The International League Against Epilepsy recognizes there are sufficient distinguishing characteristics for MTLE to be considered a distinct syndromic entity [11]. Although the two categories of TLE frequently share the same limbic semiology [11,12,13], in general, MTLE more commonly displays the epigastric, cephalic or experiential aura, loss or awareness, staring, automatisms and posturing that are typical temporal lobe seizure patterns and are the result of a seizure prominently involving the limbic structures [14]. The imaging finding that defines MTLE is atrophy and sclerosis of the hippocampus, so-called mesial temporal sclerosis (MTS) [15]. The childhood history of patients with MTLE commonly includes the presence of childhood febrile convulsions, especially of a prolonged and complicated nature [16]. Individuals with neocortical epilepsy are more likely to manifest signs related to peri-Sylvian structures such as a simple auditory hallucinations or, in the dominant hemisphere, postictal aphasia [17,18]. Although neocortical epilepsy often spreads along fibers richly connected with the mesial temporal structures manifesting the signs and symptoms of limbic involvement, which are the semiological features of MTLE as well. 

Due to the considerable overlap in semiology between the two categories of temporal lobe epilepsy, multiple noninvasive data elements must converge to localize the TLE focus to the mesial or lateral structures of the temporal lobe [19,20]. However, when patient history, seizure semiology, EEG localization, and imaging findings point to MTLE, there is a high degree of confidence in the diagnosis [21,22]. In a minority of patients, intracranial electrode monitoring may be required to investigate lateralization of the seizure onset to a temporal lobe [23] or to confirm temporal lobar localization in one hemisphere [24].

## 2. Surgery of Temporal Lobe Epilepsy

### 2.1. Temporal Lobectomy

Temporal lobectomy is a frequently a misused term that is defined as removal of the entire temporal lobe including the mesial structures. A complete removal of the temporal lobe is rarely performed today. The description of lobectomy to describe a resection should be reserved for the unique situations in which an entire anatomical temporal lobe actually is removed. The standard resections done for temporal lobe epilepsy are preferably described as an anterior temporal resection or cortico-amygdalohippocampectomy (CAH), which more accurately describes the anatomic structures that are removed.

### 2.2. Cortico-Amygdalohippocampectomy (CAH)

This is the standard temporal resection commonly performed at most epilepsy centers. It essentially corresponds to an anterior temporal neocortical resection followed by a removal of the temporal mesial structures. Variations on this approach have been described including anatomically standardized resections [24,25,26] as well as a tailored type of operation [27]. 

**Figure 1:**
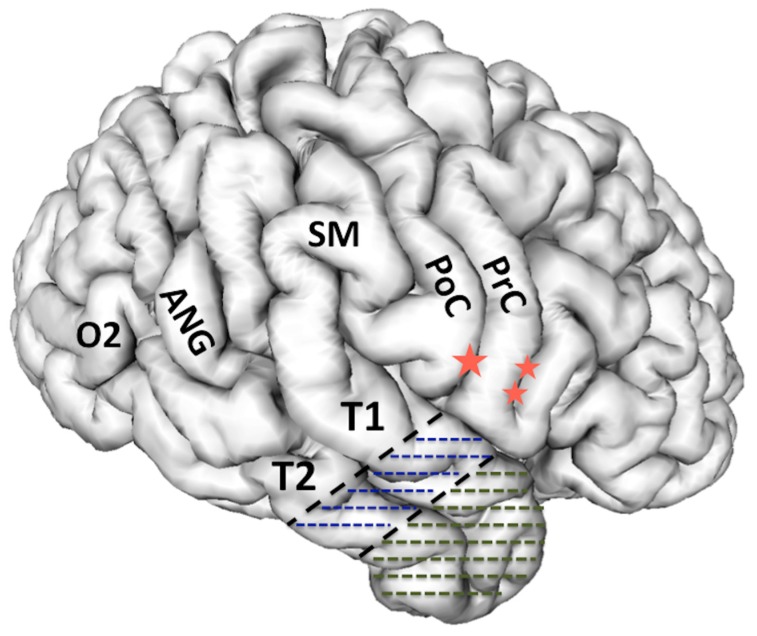
3-D reconstruction of the non-dominant right hemisphere. The dashed line illustrates the anatomical resection approach of cortico-amygdalohippocampectomy. In the non-dominant hemisphere, the resection is taken posteriorly to the level of the central sulcus along T1. In the dominant hemisphere, the T1 resection is no further posterior than the precentral sulcus in order to respect and preserve posterior language areas. Single star = central sulcus, double star = pre-central sulcus, T1 = superior temporal gyrus, T2 = middle temporal gyrus, PoC = post central gyrus, PrC = precentral gyrus, SM = supramarginal gyrus, ANG = angular gyrus, O2 = second occipital gyrus, which is the gyral continuum of T2 in the occipital lobe.

The anatomical resection approach consists, in the dominant hemisphere of the resection along the superior temporal gyrus (T1) posteriorly to the level of the pre-central sulcus, which corresponds to about 3.5–4 cm from the temporal pole. In addition, in the non-dominant hemisphere the T1 removal is delineated posteriorly by the central sulcus, which is about 4–4.5 cm from the temporal pole. Figure 1 The cortex and white matter lateral to the collateral sulcus is removed en bloc as a specimen in the first step. Next, the temporal horn is opened in order to identify the mesial temporal structures, which are disconnected and removed as described below for the selective amygdalohippocampectomy (SAH).

### 2.3. Cortico-Amygdalectomy (CA)

As the name implies, CA consists of an anterior temporal resection along with the amygdala and uncus. The remaining hippocampal formation, parahippocampus, and entorhinal cortex are spared. The lateral neocortical resection is limited posteriorly as described above for CAH. Figure 1 In the CA, the cortical resection is limited medially by the collateral sulcus thus preserving parahippocampus and entorhinal cortex. The anterior temporal horn is opened for the purpose of identifying intraventricular landmarks to confirm that the hippocampus and the adjacent parahippocampal gyrus are left intact. Opening the ventricle also facilitates removal of the amygdala and subpial emptying of the uncus. This surgical approach is most useful in patients who are at risk for a functional memory decline from removal of the hippocampal complex or have failed the ipsilateral intracarotid amobarbital test for memory. Kim, et al. identified with neuropsychological testing before and after surgery that this surgical approach avoids memory decline, yet provides a good opportunity for seizure freedom in patients with TLE when the hippocampus retains significant memory function [28,29].

### 2.4. Selective Amygdalohippocampectomy (SAH)

The SAH surgical approach is based on the understanding that the seizure focus of MTLE is confined to the mesial temporal structures. Hughlings Jackson first illustrated this concept when he described a patient with a lesion in the uncus causing psychomotor seizures (Jackson called a dreamy state), which was the first description of the role the temporal lobe structures in human epilepsy [30]. More modern experimental studies have subsequently identified the critical role of the mesial temporal structures in experimental and human TLE [31,32,33,34,35,36]. Additionally, seizure freedom results realized from many patients operated for MTLE with SAH confirms the importance of the mesial temporal and limbic structures in MTLE [35,37,38,39,40]. Because MTLE represents a sufficiently distinct pathophysiological entity, the International League Against Epilepsy has concluded that it does represent a specific epilepsy syndrome [11].

**Figure 2:**
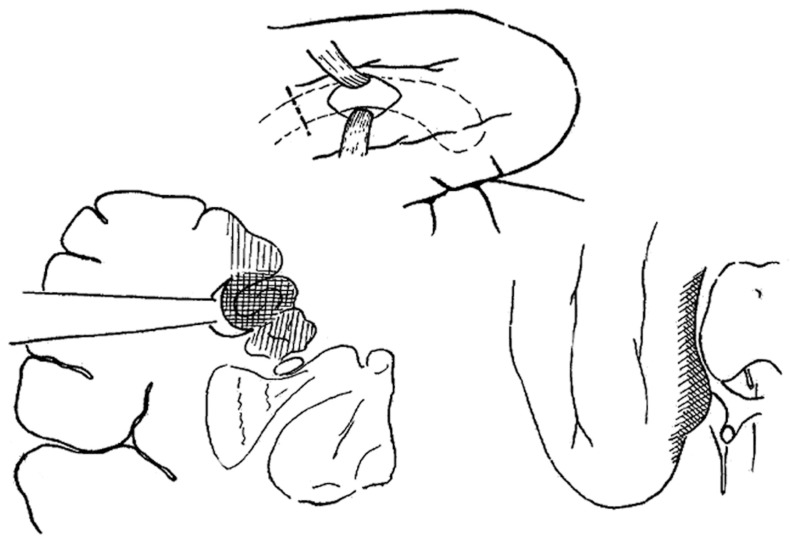
Reproduced with permission from Temporal Lobe Epilepsy: A Colloquium Sponsored by the National Institute of Neurological Diseases and Blindness, National Institutes of Health, Bethesda, Maryland, in Cooperation with the International League Against Epilepsy [45] Copyright©1959 American Medical Association. All rights reserved. In his presentation at the colloquium, Niemeyer described the convincing evidence from experimental research and clinical experience that the mesial temporal structures have a critical role in epileptogeneis of TLE. Niemeyer described a surgical technique he developed to selectively remove the mesial temporal structures via a trans-T2 trans-ventricular approach.

The SAH surgical approach was first described by the Brazilian neurosurgeon, Paolo Niemeyer in the 1950’s to treat TLE arising from the mesial temporal structures. Niemeyer described the innovative surgical technique he called “transventricular amygdala-hippocampectomy” and presented the rational for a selective approach to surgery for temporal lobe epilepsy at the International Colloquium on Temporal Lobe Epilepsy in 1957 that took place at the National Institutes of Health [41]. Figure 2 Subsequently, the importance of MTLE in medically intractable TLE has been established by reports confirming the utility of SAH for the treatment of MTLE, [38,42,43,44] and MR imaging now greatly enables the diagnosis of patients with MTLE by defining the anatomical correlate, which is hippocampal sclerosis [11,15].

Niemeyer’s description of a white matter corridor through the middle temporal gyrus (T2) to the temporal horn to selectively remove the mesial temporal structures was a very different approach than the anterior temporal resection popular at the time. In fact, research carried out in Montreal by Scoville and Milner [46] and Penfield and Milner [47], which had stressed the important role of the hippocampus in memory, as well as the earlier described Klüver-Bucy syndrome had influenced centers surgically treating epilepsy in North America to preserve the hippocampus. Concerns about memory were reflected in the first publication of a series of patients operated for TLE in which Penfield and Flanigin described hippocampus removal in only 2 of 32 patients operated [48]. However, soon after the Penfield and Flanigin publication, experimental evidence became overwhelming that the mesial temporal structures had an important role in epileptogenesis of TLE [31,32,34,49,50]. Penfield and Jasper demonstrated a few years after the Penfield and Flanigin publication that further removal of the hippocampus could convert a failed TLE surgery into a success [36]. In Britain, Murray Falconer was an early advocate of including the hippocampus in the resection for TLE. He spoke frequently about removal of the mesial temporal structures to provide the best opportunity for seizure freedom in TLE, and Falconer identified the importance of mesial temporal sclerosis (he coined the term) in the pathogenesis of TLE [51]. William Feindel in Montreal demonstrated an important role of the amygdala in epileptogenesis and in generation of the typical automatism semiology of TLE [52]. 

**Figure 3:**
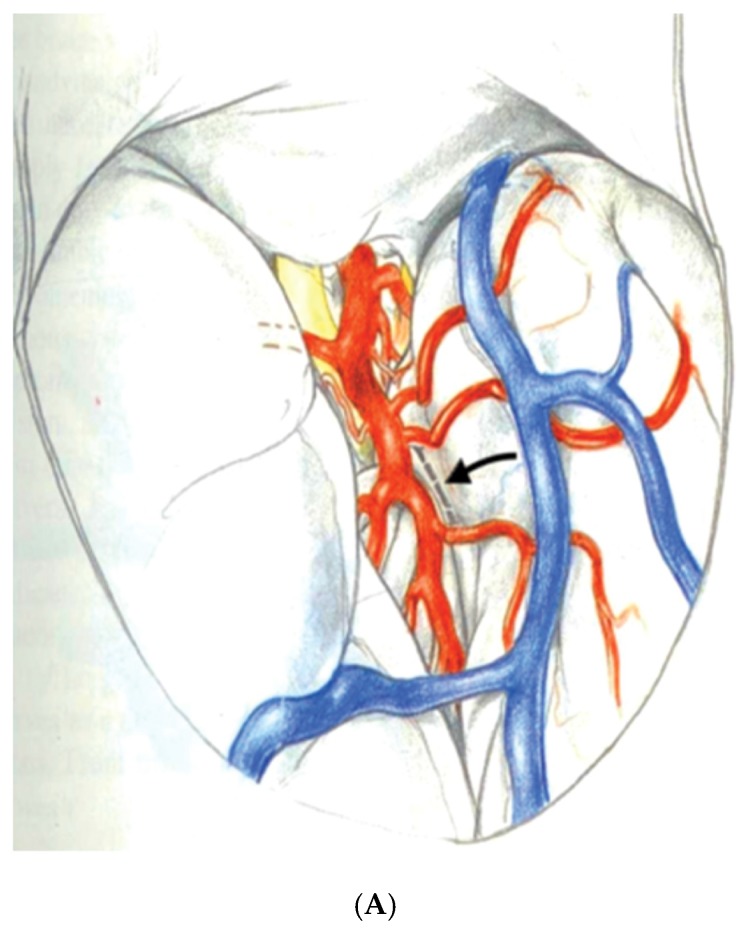

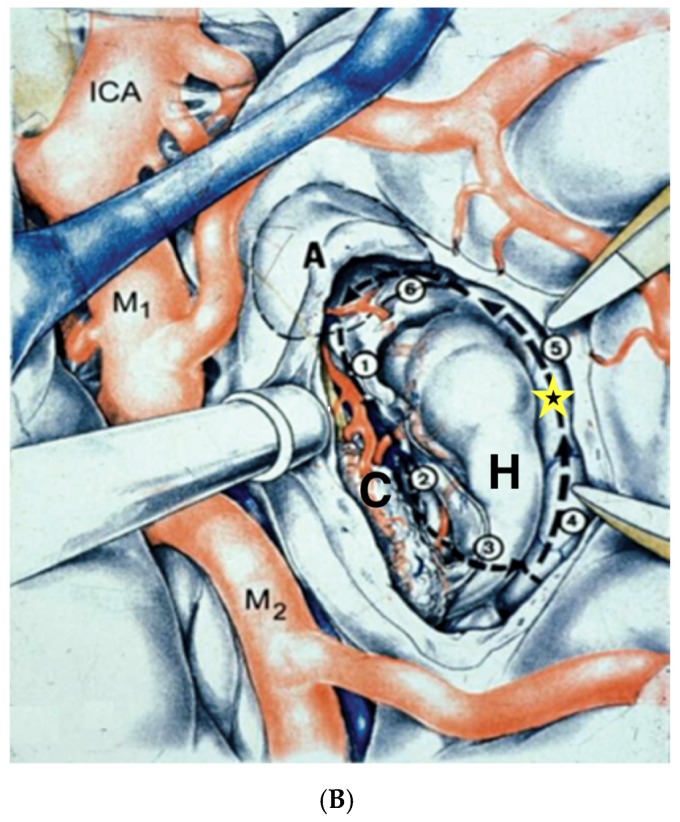
Reprinted and Modified by permission from Springer Nature: Adv Tech Stand Neurosurg, Yaşargil MG, Teddy PJ, Roth P. Selective amygdalohippocampectomy: operative anatomy and surgical technique. Copyright, 1985 [55]. (**A**) The Sylvian fissure is opened widely to expose the inferior delineation of the insula called the circular sulcus with the planned white matter incision marked in a dashed line between two middle cerebral opercular temporal arteries; (**B**) Surgeon’s view into the opened ventricle illustrating the choroid plexus (C), hippocampus (H), collateral eminence (star), middle cerebral vessels (M1 and M2), and amygdala (A). Numbers represent steps in the extra-pial removal of mesial temporal structures: coagulation and division of the hippocampal vessels (steps 1 and 2), disconnection of the hippocampal body from the hippocampal tail (step 3), opening the collateral eminence to empty and remove the parahippocampus (steps 4 and 5), and remove uncus and amygdala (step 6).

Yasargil, et al. generated considerable interest in SAH for MTLE after the description of a selective surgical approach using the trans-Sylvian fissure route to remove the mesial temporal structures [53]. In the technique described by Yasargil, the Sylvian fissure is opened to expose the inferior aspect of the circular sulcus of the insula. An incision in the circular sulcus between middle cerebral temporal opercular arteries and dissection through the white matter of the temporal stem will open the temporal ventricular horn providing sufficient exposure to identify then remove the hippocampal formation and amygdala. Figure 3A,B An advantage of this surgical approach to SAH is to minimize white matter dissection required to access the mesial temporal structures as compared with Niemeyer’s technique. The disadvantages of the trans-Sylvian approach are mostly related to the demanding technical requirements of widely opening the Sylvian fissure and working between the middle cerebral vessels within the fissure. Manipulation of the middle cerebral arteries during surgery in addition to the extra-pial resection of the hippocampus are technique related aspects of the procedure that may increase the risk of vascular injury [54].

**Figure 4:**
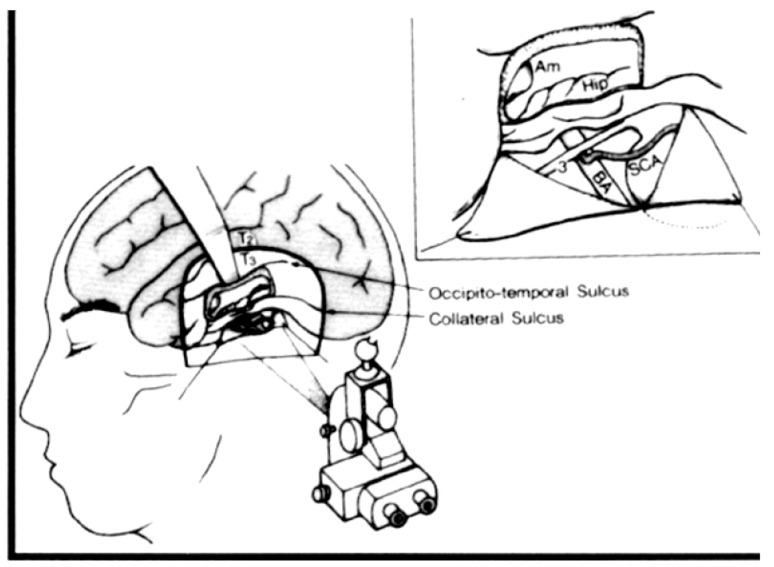
Reproduced with permission from: Figure 1. Tomokatsu Hori et al. Subtemporal Amygdalohippocampectomy for Treating Medically Intractable Temporal Lobe Epilepsy. *Neurosurgery* (1993) 33 (1): 50–57, [56]. Published by Oxford University Press on behalf of the Congress of Neurological Surgeons. Hori developed the subtemporal approach to SAH. The illustration shows a retractor elevating T3 (inferior temporal gyrus). This approach requires a gyrectomy of the fusiform gyrus to obtain access to the mesial structures. Hori, et al. also demonstrated in the inset figure that incising and reflecting the tentorium benefits accessing the mesial temporal structures for removal.

Hori contributed importantly to the SAH literature by describing a subtemporal amygdalohippocampectomy technique to perform SAH [56]. This approach includes at least partial removal of fusiform gyrus in order to gain access to the parahippocampal gyrus and mesial structures via a subtemporal route. Figure 4 Hori’s original description was extra-pial resection of the mesial temporal structures but later he modified the approach to a subpial resection technique. To minimize retraction on the temporal lobe, Hori drilled down the roof of the external auditory meatus. A preoccupation with the subtemporal approach is retraction injury of the anastomotic vein of Labbé, which Hori was able to successfully preserve using techniques described in the skull base surgery literature [57]. The advantages of the subtemporal approach that Hori described were preservation of visual fields, he identified no superior quadrantanposia after surgery, and the temporal lobe is not disconnected in contradistinction to Yasargil’s trans-Sylvian approach that requires dissecting through the temporal stem to access the mesial temporal structures. The disadvantages of the subtemporal approach are related mostly to the extended resection of the fusiform gyrus that is not required in other SAH approaches as well as an inherent risk of injury to the anastomotic draining vein of Labbé from elevating the temporal lobe with a retractor, which is a particular concern in the dominant temporal lobe.

Over many years now, a transcortical approach through the second temporal gyrus has been used by the author similar to the approach originally described by Niemeyer [58,59]. SAH achieves to provide the best possible outcomes of seizure freedom by limiting the resection to the epileptic focus and sparing from resection normal structures that are not primarily a part of the seizure focus. At centers with experience in SAH, the results on seizure freedom are the same as the larger anterior temporal resection or cortico-amygdalohippocampectomy (CAH) [24,25,26,27,28,29,35,38,44,56,58,60]. The neuropsychological results of SAH appear to be improved compared to CAH although this conclusion is not shared by all investigations on the topic [42,61,62]. However, the objective of SAH to spare brain tissue not involved with the seizure focus is an important goal of epilepsy surgery in general.

## 3. Surgical Technique of SAH

The scalp and bone exposure for SAH can range from a frontotemporal craniotomy to a keyhole minimal access approach. Electrocorticography (ECOG) requires the larger craniotomy to fit the surface electrodes in the bony opening. However, if ECOG is not needed, keyhole minimal access is the preferred approach. 

The traditional frontotemporal craniotomy is exposed via a curvilinear “?” mark scalp incision that starts at the zygoma, extends posterior just to the back of the ear, up to the superior temporal line, and anterior to the hair line. The temporalis muscle is reflected anteriorly with scalp. Exposing the root of the zygoma is important to gain adequate exposure to the floor of the middle fossa. After elevation of the frontotemporal bone flap, a craniectomy of the temporal bone is required to extend the bone opening to the zygoma inferiorly and to the anterior extent of the middle fossa. After the dura is reflected away from the temporal lobe, important sulcal and gyral landmarks, such as the Sylvian fissure, superior temporal gyrus (T1), and the central sulcus are confirmed with image guidance. 

**Figure 5:**
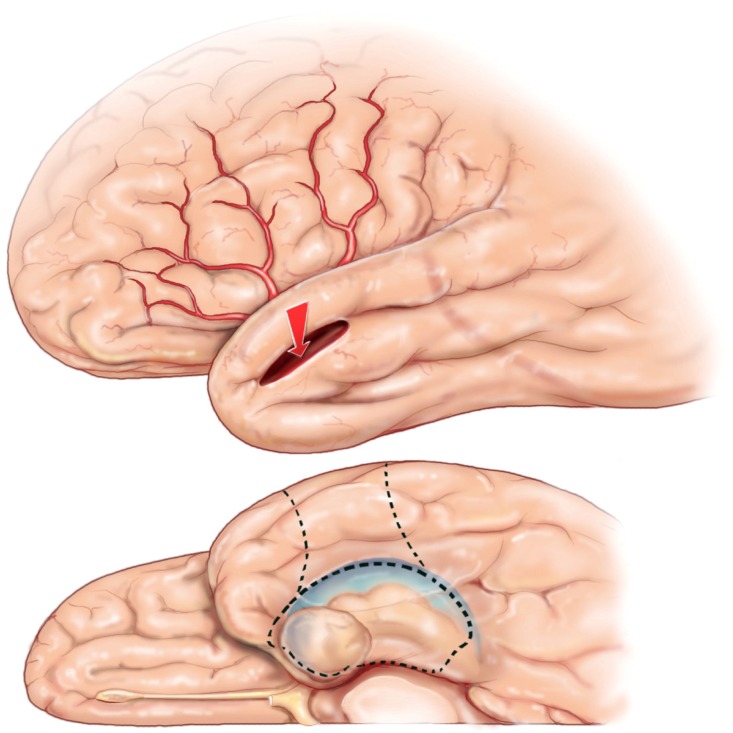
SAH corticectomy of about 2.5 cm is made along T2 just below the superior temporal sulcus. A white matter corridor is fashioned that follows the superior temporal sulcus down to the temporal horn, which is opened to visualize directly the mesial temporal structures.

**Figure 6:**
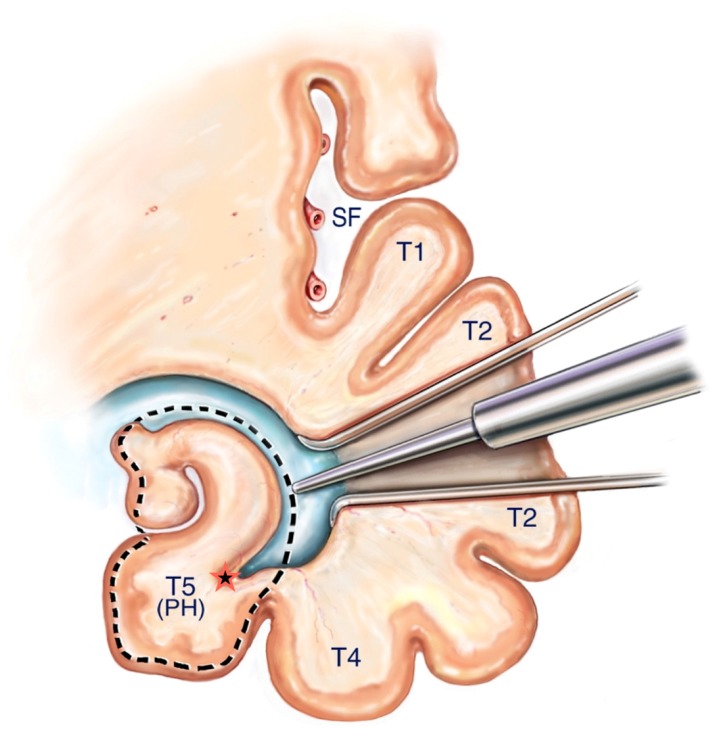
Reprinted from Journal of Clinical Neuroscience 17 (9), Boling W, Minimal access keyhole surgery for mesial temporal lobe epilepsy, 1180–1184, Copyright 2010, with permission from Elsevier, [59]. Coronal view of the temporal lobe and nearby structures. A retractor is placed along the white matter corridor after opening the ventricle to fully expose its contents. The first step in the SAH is to enter the lateral ventricular sulcus (star) that lies between the bulges into the ventricle of the hippocampus and collateral eminence in order to empty the parahippocampus in a subpial fashion. Dotted line illustrates mesial temporal structures to be removed in this view, namely the hippocampal complex and parahippocampus. SF = Sylvian Fissure, T1= superior temporal gyrus, T2 = middle temporal gyrus, T4 = Fusiform gyrus, T5(PH) = parahippocampal gyrus.

A corticectomy along the superior aspect of the second temporal gyrus (T2) is made to begin the corridor to the mesial temporal structures. A 2 to 3 cm corticectomy provides sufficient working space. Figure 5 The posterior extent of the corticectomy is limited by the central sulcus in the non-dominant hemisphere and by the precentral sulcus in the dominant hemisphere. Image guidance is sufficient to localize precentral and central sulcus, which corresponds to about 3.5 cm and 4.5 cm from the temporal tip, respectively. The author has found that using an ultrasonic aspirator at the lowest settings of aspiration and amplitude to be the best subpial dissection and aspiration tool. A corridor is fashioned down to the temporal horn that is opened, which is typically 3–4 cm deep from the cortical surface. The corridor trajectory parallels the superior temporal sulcus, which points to the temporal horn and stops just short of it. A retractor is inserted once the temporal horn is opened to facilitate fully exposing the mesial temporal structures by opening the ventricle from its anterior most tip to the posterior limit of the corridor Figure 6.

**Figure 7:**
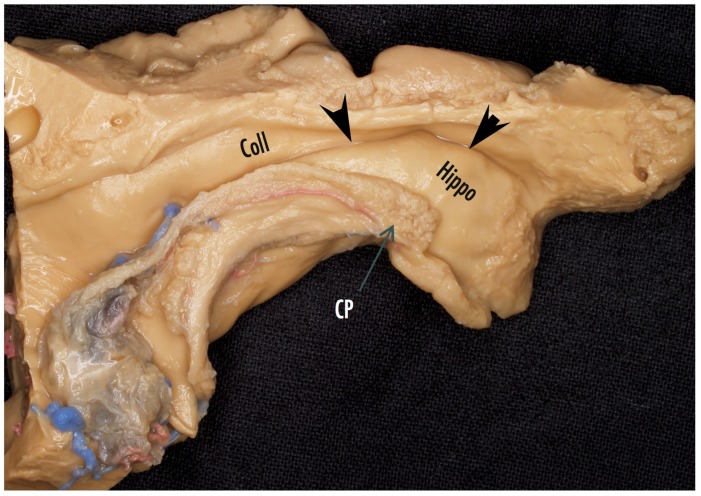
Cadaver dissection of the mesial temporal lobe cut longitudinally along the temporal horn to illustrate the mesial temporal structures. Coll = collateral eminence, arrowheads point to the lateral ventricular sulcus, Hippo = head of the hippocampus, CP = choroid plexus.

The resection of the mesial temporal structures is subpial preferably using the ultrasonic aspirator at its lowest settings of aspiration and amplitude, which is an excellent tool for subpial resection. There are several key intraventricular landmarks identified in the mesial temporal region that guide the SAH surgery. The lateral ventricular sulcus lying between the two bulges into the ventricle of the hippocampus and the collateral eminence (see Figure 6 and Figure 7) is the entry into the parahippocampal gyrus that is emptied subpially as the first surgical maneuver. The P2 segment of the posterior cerebral artery is usually identified bulging into the parahippocampus, which fills the space between the collateral sulcus and hippocampal sulcus. The hippocampus can now be retracted laterally into the space created by removing the parahippocampus in order to aspirate the fimbria from the underlying pia. The hippocampus is next removed en bloc by disconnection from the hippocampal tail posteriorly, which enables the hippocampus to be elevated and separated from its vascular pedicle, the hippocampal sulcus, by teasing and separating vessels from the hippocampus or by coagulation and division. Additional hippocampus can be aspirated and removed back to the typical posterior limit, which corresponds to the level of the midbrain tectum, a landmark visualized readily with image guidance. 

**Figure 8:**
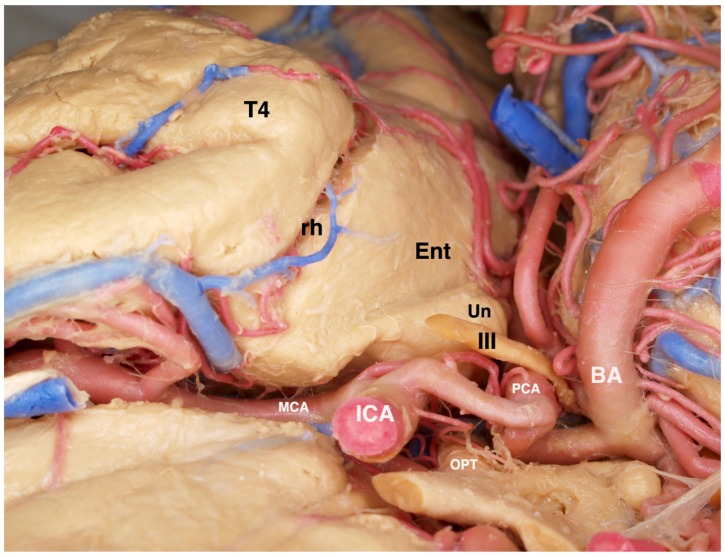
View of a fixed and injected brain from the orbital frontal surface looking posteriorly. The normal relationships of structures lying adjacent to the mesial temporal lobe are visualized. The 3rd cranial nerve is normally abutting the pia of the anterior uncus. The posterior cerebral artery can be recognized along its course beneath the transparent pia of the uncus and the parahippocampus. The close association of the ICA and its bifurcation with the uncus and amygdala are illustrated. T4 = fusiform gyrus of the temporal lobe, rh = rhinal sulcus, Ent = entorhinal cortex (most anterior extent of the parahippocampus), Un = uncus, III = oculomotor cranial nerve, ICA = internal carotid artery, PCA = posterior cerebral artery, OPT = optic chiasm, BA = basilar artery, MCA = middle cerebral artery.

The anterior extent of the SAH resection includes the entire uncus that should be completely emptied subpial. The uncus is composed mostly of the hippocampus and dentate gyri that have curved back on themselves to create a hook-like appearance. In very close proximity to the transparent pia of the uncus, cranial nerve III and the P1 segment of the posterior cerebral artery are typically visualized beneath the pia lying in their cistern. Figure 8 The amygdala, which contributes to the dorsomedial portion of the uncus, called the semilunar gyrus, is either aspirated completely or dissected around and removed en bloc. The choroid plexus is an important landmark to identify early and repeatedly refer to because it limits the mesiosuperior resection extent.

**Figure 9:**
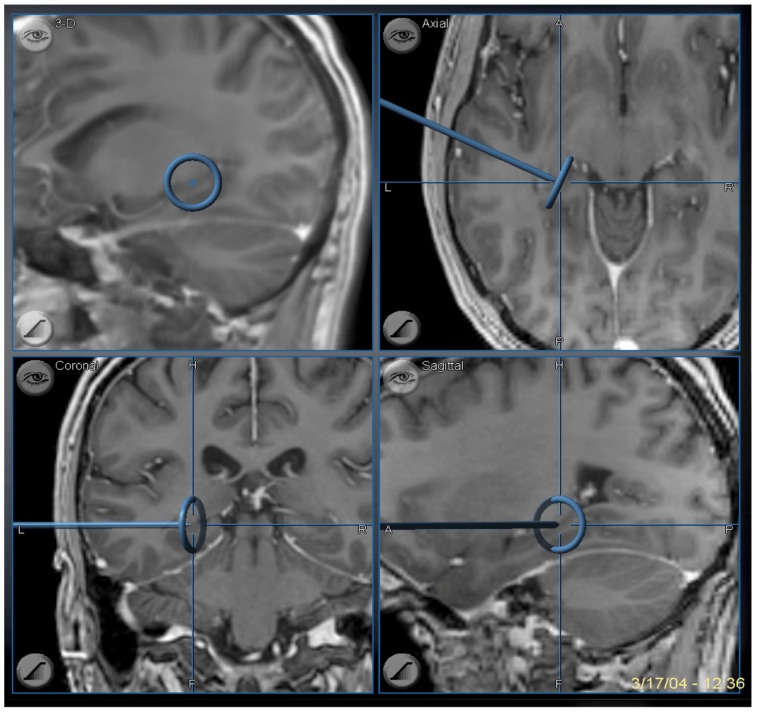
Image guidance view of the navigation pointer at the posterior extent of the hippocampal removal, which corresponds to the level of the midbrain tectum.

The completion of the SAH corresponds to removal of the mesial temporal structures found between the choroid plexus and collateral sulcus that includes the uncus, amygdala, hippocampus, parahippocampus, and fimbria. All of which are removed by keeping the underlying pia intact. The anterior limit is to the anterior extent of the uncus at the tentorial incisura and the posterior limit is back to the level of the midbrain tectum (Figure 9).

## 4. Key Hole Approach in SAH

Minimal access surgery has potential to benefit patients by providing more rapid recovery and improved patient outcomes compared with traditional larger surgical incisions and exposures [60,63,64]. The minimal access (or keyhole) approach lends itself quite well to SAH using either the trans-middle temporal gyrus or subtemporal approaches since the necessary maneuvers required to remove the mesial temporal structures can be accomplished with relative ease through a small scalp incision and bony opening [29,59,60,65]. The SAH approach was envisioned and developed in order to spare from resection cerebral tissue that is not a part of the primary seizure focus. Therefore, minimizing the scalp and bony opening to accomplish SAH represents a logical progression from the larger more traditional scalp incision and craniotomy exposure. In individuals with clear-cut MTLE who are candidates for SAH, the keyhole, minimal access approach is preferred. Because the exposure is too limited to confidently recognize sulcal and gyral landmarks, keyhole SAH should be performed with the aid of image guidance. 

**Figure 10:**
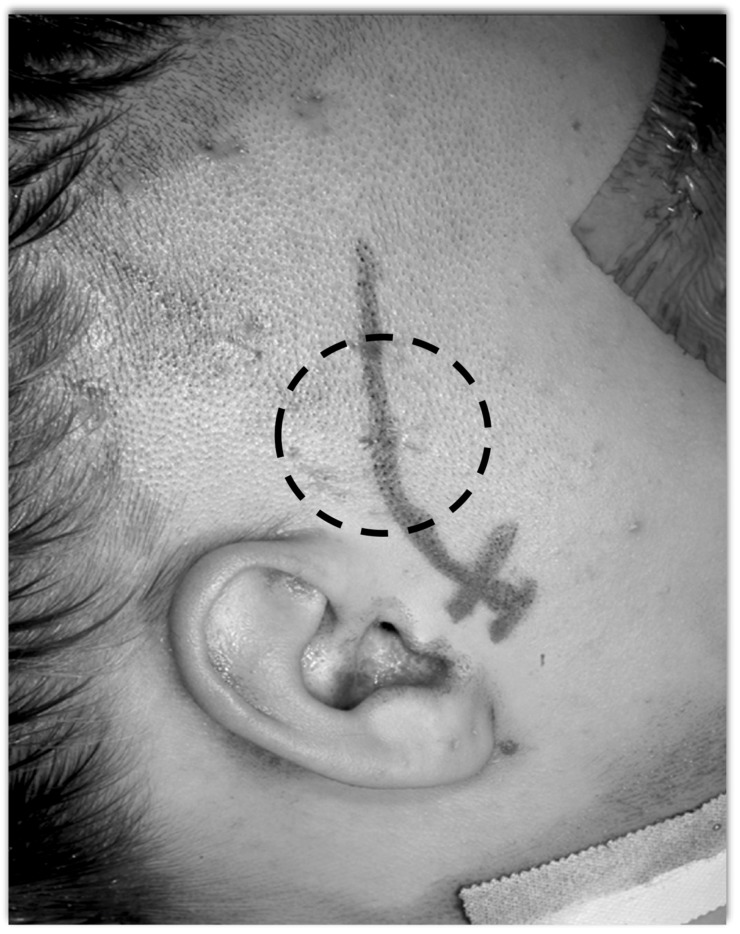
The keyhole minimal access approach benefits the patient with smaller skin incision and cranial opening. A slight curve in the scalp incision helps exposure and reduces retraction forces on the temporalis muscle compared with a straight linear incision.

**Figure 11:**
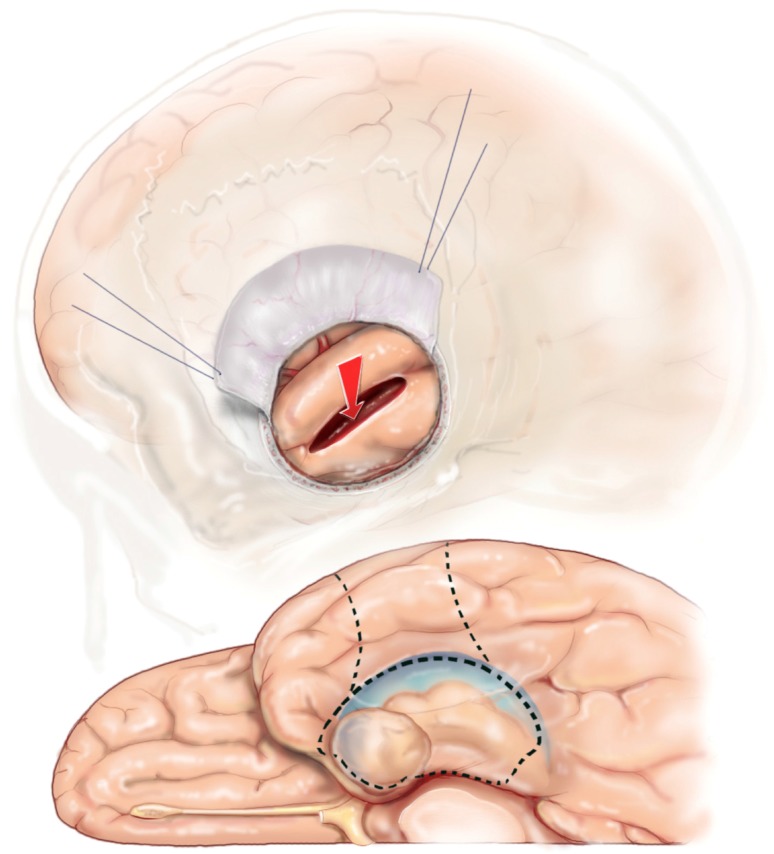
Reprinted from Journal of Clinical Neuroscience 17 (9), Boling W, Minimal access keyhole surgery for mesial temporal lobe epilepsy, 1180–1184, Copyright 2010, with permission from Elsevier, [59]. The corticectomy and trans-T2 trans-ventricular approach to resection of the mesial temporal structures is identical to the approach via a standard scalp incision and cranial opening. The exposure accomplished with a keyhole access approach is more than adequate to perform the maneuvers required to complete the SAH.

The scalp incision is curvilinear starting at the zygoma. Figure 10 The temporalis muscle is split and held open with hooks or a self-retaining retractor. The bone opening is a silver dollar sized craniotomy plus additional craniectomy anterior and inferior centered over the second temporal gyrus and the planned cortical incision. Figure 11 The keyhole and non-keyhole SAH approach to the mesial temporal structures are essentially identical. Image guidance is critical to confirm the corticectomy is positioned properly in the middle temporal gyrus anterior to the central sulcus in the non-dominant hemisphere and anterior to the pre-central sulcus in the dominant hemisphere. 

## 5. Percutaneous Ablation Approaches in SAH

For the past many decades there has been interest in the stereotactic insertion of a probe to target the mesial temporal structures for thermal ablation in patients with TLE. The early efforts used stereotactic radiofrequency thermal ablation techniques that were also being used at the time to lesion the thalamus to treat movement disorder, the Gasserian ganglion to eliminate the pain of trigeminal neuralgia, and other neurological disorders that could be treated stereotactically. The amygdala was solely targeted in the initial reports of stereotactic ablation in the treatment of epilepsy. The reports of Schwab, et al. [66] and Narabayashi, et al. [67] described approximately 30% seizure free patients after stereotactic amygdalectomy for TLE. Flanigin and Nashold, both former fellows of the Montreal Neurological Institute, were able to demonstrate the feasibility of multiple stereotactic ablations to expand the volume of mesial temporal structures treated to include the hippocampus along with the amygdala, as well as bilateral amygdalectomies [68]. However, more reports of amygdalectomy and hippocampectomy treated with stereotactic radiofrequency probes continued to be disappointing in regards to seizure free rates and did not approach the seizure free successes achieved with anterior temporal lobe resection [69,70,71,72]. The early efforts at stereotactic ablation of the mesial temporal structures to treat intractable TLE were largely hampered by the challenges of targeting the mesial temporal structures using the methods of the time; stereotactic targets were defined by imaging with angiogram and pneumoencephaolgraphy or by measurements made from the AC-PC line. Also, the early stereotactic frames were best suited for orthogonal trajectories, which made treatment along the longitudinal axis of the mesial temporal structures a more arduous treatment plan.

With the advent of CT and MR imaging, the mesial temporal structures could be directly and more accurately targeted for stereotactic treatment. Parrent and Blume reported the experience of stereotactic amygdalohippocampectomy for TLE with the use of modern MR imaging [73]. The authors described outcomes from patients with TLE and evidence of exclusively ipsilateral MTS in 11 out of 19 patients treated. Surgery was performed using a stereotactic headframe with radiofrequency lesioning of the amygdala and hippocampus. Postoperative MRI identified the best results were in patients with more complete stereotactic ablations involving the entire amygdala and measuring 15–34 mm along the hippocampus. In this group of patients, 9 out of 15 had favorable results defined as greater than 90% reduction in seizure frequency or seizure freedom. Only 1 out 5 patients with incomplete ablation of the amygdala and hippocampus had a favorable result. Parrent and Blume were able to demonstrate the improved results of stereotactic ablation surgery using modern imaging to target the thermal probe, and that more complete treatment of the amygdala and hippocampus produced better results of seizure freedom and seizure frequency reduction. 

Three additional advances have enabled further improvement in the technique of stereotactic ablation surgery of TLE. One is frameless stereotaxy or image guidance. Now that stereotactic surgery can be performed without the traditional stereotactic headframe in place, neurosurgeons have considerably more freedom to plan non-orthogonal trajectories to targets that were more difficult or even impossible to accomplish previously. Another advance is the development of low cost fiber-optic lasers for thermal ablation. The radiofrequency ablation probe has not been able to be manufactured to allow use in the MRI magnet. Using the laser ablation technology, only the non-ferromagnetic fiber optic cable is in the MRI environment. Therefore, the thermal treatment can be delivered with the patient in the MRI to confirm the anatomy treated and the thermal dose delivered, which segues to the third advance, the ability to create a thermal map using normal MRI acquisition protocols. Several measurable MRI variables are affected by temperature in a linear relationship and can be used to construct a thermal map, such as diffusion coefficient [74], chemical shift, T1 relaxation [75], magnetization transfer, proton density and proton resonance frequency (PRF). The excellent linearity and temperature dependence of the PRF in relation to most all tissue types have made PRF-based phase mapping methods the preferred choice for thermal mapping and this acquisition protocol is available on most all modern MRI units. 

There are 2 FDA approved laser thermal ablation devices that overlay anatomy with a thermal map, NeuroBlate (Monteris Medical, Plymouth, MN) and Visualase (Medtronic, Minneapolis, MN). Both devices use a pulsed laser thermal source. The probe, which consists of a fiber-optic cable to deliver the laser plus a cooling system, is inserted stereotactically to the desired target in the operating room. Then the actual thermal ablation treatment is performed in the MRI magnet. There has been considerable renewed interest in the use of thermal ablation for the treatment of intractable MTLE, and the fiber-optic laser technologies have improved the ability to target the mesial temporal structures and assess the treatment delivered essentially real-time. 

The fiber-optic laser thermal probe is typically inserted in a posterior to anterior trajectory to allow treatment of the mesial temporal structures along the longitudinal axis. Multiple thermal treatments are delivered as the fiber-optic probe is pulled back until a sufficient volume of mesial temporal structures are ablated. One of the challenges of treatment planning is to insert the probe in such a trajectory that the uncus and hippocampal head can be completely ablated. Jermakowicz, et al. found lateral trajectories that did not include the uncus and hippocampal head in the ablation treatment were significantly correlated with persistent disabling seizures [76]. Overall the laser ablation of mesial temporal structures has been shown to be a safe minimally invasive alternative to craniotomy surgery of MTLE. Although the data thus far point to reduced odds of seizure freedom in patients treated with laser thermal ablation versus craniotomy approaches to resect the mesial temporal structures [77]. 

## 6. Patient Outcomes from SAH 

The success of SAH to achieve seizure freedom is dependent on the accurate diagnosis of MTLE lateralized to the side of surgery and the successful removal or disconnection of the seizure focus at surgery. At centers with expertise in the diagnosis of MTLE, the seizure freedom rates after SAH are the same as anterior temporal resection [29,35,59,60,65,78,79]. To date a randomized controlled study to evaluate and compare the surgical approaches of SAH and ATL for the treatment of MTLE has not been undertaken. The literature up to now demonstrates no clear difference in ability to become seizure free in patients with surgery of SAH or ATL for MTLE (Table 1).

One potential advantage of SAH is that cognitive function may be improved in patients with more limited temporal lobe resections. This topic was reviewed by Schramm [94] who found the literature lacking consistency on reporting of crucial study variables such as MRI confirmation of extent of resection and studies were mostly retrospective reports. However, Schramm concluded from the available literature that “in summary there is considerable evidence for somewhat better neuropsychological results with SAH, although this was not consistently found.” An analysis of studies published from 1992 to 2018 supports Schramm’s conclusion that in general there tends to be more neuropsychological deficits in ATL than SAH surgery, although the heterogeneity of studies and the lack of a randomized controlled study comparing the two surgical techniques makes firm conclusions about neuropsychological impact impossible to make (Table 2).

An additional outcome parameter of considerable importance to the patient is the ease and speed of recovery from brain surgery. SAH has an opportunity to provide patients shorter operative time, quicker recovery, and reduced length of stay in the hospital particularly if surgery is performed using minimal access or key hole approaches [59,105]. Reduced hospital length of stay is a marker of both quicker patient recovery and a more cost effective operation. Boling identified significantly reduced operative time and shorter hospital length of stay in patients who underwent key hole craniotomy versus a standard craniotomy approach for SAH [59].

## 7. Conclusions

Patients with medically intractable MTLE have an excellent opportunity for seizure freedom from resection surgery that can be a standard anterior temporal removal or selective amygdalohippocampectomy. The potential advantage of the SAH is a selective removal of the seizure focus sparing temporal lobe regions that are not the actual epileptogenic zone. SAH, especially minimal access approaches, provide direct advantages of quicker patient recovery from surgery, and evidence points to a probable cognitive benefit compared with standard temporal resections.

## Figures and Tables

**Table 1:** Studies comparing ATL with SAH for outcomes of seizure freedom and reducing seizure frequency.

Renowden et al. (1995) [80]	Same outcome for two types of SAH
Arruda et al. (1996) [81]	Similar
Pauli et al. (1999) [82]	Similar
Clusmann et al. (2002) [83]	Similar
Clusmann et al. (2004) [40]	Better in ATL in children + adolescents
Lutz et al. (2004) [40]	Same outcome for two types of SAH
Paglioli et al. (2006) [84]	Similar
Tanriverdi, et al. (2007) [85]	Similar
Bate et al. (2007) [86]	Better in ATL in children + adolescents
Tanriverdi, et al. (2010) [87]	Similar
Sagher, et al. (2012) [78]	Similar
Wendling, et al. (2013) [88]	Similar
Bujarski, et al. (2013) [89]	Similar
Josephson, et al. (2013) [90]	Better outcome in ATL
Hu, et al. (2013) [91]	Better outcome in ATL
Nascimento, et al. (2016) [92]	Similar although ATL associated with more complications
Schmeiser, et al. (2017) [93]	No difference in three types of SAH nor in SAH versus ATL
Foged, et al. (2018) [94]	No difference at 1 year and 7 years after surgery

**Table 2:** Studies comparing temporal lobe surgery of ATL with SAH for neuropsychological outcome.

Goldstein, et al. (1992) [95]	No difference when using global memory test
Goldstein, et al. (1993) [96]	SAH short-term beneficial effect on memory
Wolf et al. (1993) [97]	No difference
Renowden et al. (1995) [80]	SAH better in verbal IQ and nonverbal memory
Helmstaedter et al. (1996) [98]	Immediate recall better in SAH
Jones-Gotman (1997) [42]	Similar deficits in learning + retention tasks in seizure free patients
Pauli et al. (1999) [82]	SAH better for verbal memory
Helmstaedter et al. (2002) [99]	SAH has advantage over ATL in long-term follow-up (2–10 years)
Clusmann (2004) [40]	SAH in adults: higher rate of improvement + lower rate of deterioration in overall neuropsychological score
Hader et al. (2005) [100]	No difference
Morino et al. (2006) [101]	SAH better memory function
Paglioli et al. (2006) [84]	SAH better for verbal memory score (30% deterioration in both groups)
Tanriverdi, et al. (2007) [85]	SAH less decline for verbal memory
Helmstaedter et al. (2008) [102]	SAH better for R-sided resection, ATL better for L-sided resection for material-specific memory
Tanriverdi, et al. (2010) [87]	Worse verbal IQ after SAH
Sagher, et al. (2012) [78]	No difference
Bujarski, et al. (2013) [89]	No difference, except more post-surgical paranoia after ATL
Wendling, et al. (2013) [88]	Worse memory after ATL
Boucher, et al. (2015) [103]	Worse after ATL on immediate recall of Logical Memory subtest of Wechsler Memory Scales. Delayed recognition trial of Rey Auditory Verbal Learning Test worse after SAH
Nascimento, et al. (2016) [92]	No difference
Gül, et al. (2017) [104]	No difference
Schmeiser, et al. (2017) [93]	No difference
Foged, et al. (2018) [94]	SAH better than ATL verbal memory in left hemisphere surgery only
